# Critical evaluation of methodology in “Experiences of insomnia among older people living in nursing homes a qualitative study”

**DOI:** 10.1080/17482631.2025.2522442

**Published:** 2025-06-27

**Authors:** Milad Kazemi Najm, Nasrin Imanifar

**Affiliations:** a Student Research Committee, Hamadan University of Medical Sciences, Hamadan, Iran; b Instructor and Faculty Member of the Department of Nursing, Khor.C. Islamic Azad University, Khorramabad, Iran; c Student Research Committee, Nursing and Midwifery Faculty, USERN Office, Lorestan University of Medical Sciences, Khorramabad, Iran

**Keywords:** Qualitative research, methodology critique, nursing homes, insomnia, content analyzes, older people

## Abstract

**Introduction:**

This letter critically evaluates the methodology of Eva Hjort Telhede’s qualitative study exploring insomnia experiences among nursing homes. While the study contributes valuable insights into subjective sleep challenges, its methodological rigour warrants scrutiny to inform future research.

**Methods:**

The study employed a qualitative descriptive design with semi-structured interviews (*n* = 19 participants) and inductive content analysis. Data collection occurred in nine Swedish nursing homes, with purposive sampling based on insomnia criteria (ICD-10) and cognitive competence (S-MMSE ≥20). Analysis followed Graneheim and Lundman’s qualitative content analysis framework.

**Results:**

Key methodological strengths included purposive sampling, data saturation, and reflexive practices. Limitations identified were single-researcher bias, lack of intercoder reliability checks, gender imbalance (4 men, 15 women), and exclusion of variables such as cognitive diagnoses and medication use. Environmental factors (e.g. noise and lighting) were self-reported without objective validation, and contextual transferability was constrained by limited demographic diversity.

**Discussion:**

The reliance on a single coder and absence of triangulation may compromise the depth of thematic analysis. Recommendations include: (1) multi-researcher collaboration to enhance credibility; (2) inclusive sampling of residents with dementia; (3) mixed-methods designs integrating objective sleep measures; and (4) staff training in sleep hygiene to address institutional barriers. Strengthening methodological transparency and addressing contextual factors could improve future interventions for insomnia in nursing homes.

## Dear Editor in chief,

Professor Henrika Jormfeldt

We read with interest the recent article by Eva Hjort Telhede (2025) exploring the experiences of insomnia among older people in nursing homes. This qualitative study provides valuable insights into the multifaceted nature of sleep disturbances in this vulnerable population (Hjort Telhede, 2025). Below, I offer a critical evaluation of the research, highlighting its strengths, limitations, and implications for future work.

### Strengths

The study’s qualitative design effectively captures the subjective lived experiences of insomnia, emphasizing the emotional, psychological, and environmental dimensions often overlooked in quantitative approaches. By employing semi-structured interviews and inductive content analysis, the author adeptly identifies two overarching categories—“Valuing good sleep” and “Disturbing influence on sleep”—with subcategories that align with existing literature on insomnia in older adults (e.g., anxiety, environmental noise, and social isolation) (Hjort Telhede, 2025). The purposive sampling strategy, grounded in ICD-10 criteria and cognitive competence (via S-MMSE), strengthens the validity of participant selection, ensuring relevance to the study’s aims.

The inclusion of direct participant quotes (e.g., “If you don’t do anything during the day, it’s boring…”) enriches the findings, offering authentic voices to contextualize the data. Furthermore, the author’s reflexive approach—acknowledging potential biases and refining interview questions through a pilot test—enhances methodological transparency.

### Limitations and areas for improvement

Despite its merits, the study has notable limitations.

#### Single researcher bias

The sole involvement of the author in data collection, analysis, and interpretation introduces potential bias. Lack of triangulation (e.g., peer debriefing, multiple coders) limits the rigour of theme identification. As noted in the study’s limitations, this could compromise the depth of analysis (Malterud, 2001).

#### Sample demographics

The sample includes only four male participants (21%), which may limit generalizability given documented gender differences in sleep patterns and insomnia prevalence (Chu et al., 2022). Future studies should aim for balanced representation.

#### Exclusion of key variables

The exclusion of residents with formal dementia diagnoses or detailed medication histories weakens the analysis of how cognitive impairment or polypharmacy might mediate sleep disturbances. These factors are critical in nursing home populations (Laver et al., 2020).

#### Environmental factors

While residents highlighted environmental disturbances (e.g., noise and lighting), the study relied solely on self-reports. Objective measures (e.g., noise sensors and actigraphy) could validate these claims and strengthen findings (Ogilvy Dunstan et al., 2025).

#### Lack of intercoder reliability

The absence of intercoder reliability checks undermines the trustworthiness of the analysis. Independent coding by multiple researchers would enhance confirmability (Elo & Kyngäs, 2008).

#### Contextual transferability

Findings are context-specific to Swedish nursing homes. The lack of data on staff-to-resident ratios, care routines, or facility size limits transferability to other cultural or institutional settings (Jung et al., 2022).

### Discussion and recommendations

The discussion adeptly links findings to prior research, such as the role of environmental factors (e.g., noise and lighting) in sleep quality (Yang et al., 2022). However, the authors could have engaged more critically with conflicting evidence—for example, studies suggesting that structured daytime activities improve sleep in nursing home residents (Kim & Yoon, 2020), which contrasts with the current finding that reduced activity exacerbates insomnia.

The conclusion’s call for person-centred care and environmental modifications is well founded. However, the recommendations remain broad. Future work could explore targeted interventions, such as staff training in sleep hygiene (Čáp et al., 2024) or personalized light therapy to regulate circadian rhythms (Kim & Yoon, 2020). Non-pharmacological strategies like cognitive behavioural therapy for insomnia (CBT-I) or weighted blankets—referenced in the author’s prior work (Telhede et al., 2024)—deserve further empirical attention.

### Recommendations for future research

#### Triangulation

Future studies should involve multiple researchers in coding and analysis to mitigate bias. Peer debriefing and audit trails could further enhance rigour (Polit & Beck, 2008).

#### Inclusive sampling

Expand recruitment to include residents with mild cognitive impairment or dementia, as these groups are often underrepresented yet highly vulnerable to insomnia.

#### Mixed-methods approach

Combine qualitative insights with objective sleep measures (e.g., actigraphy) and environmental assessments (e.g., noise levels) to contextualize self-reports.

#### Intervention studies

Explore non-pharmacological interventions (e.g., weighted blankets, cognitive behavioural therapy for insomnia [CBT-I], or light therapy) to address the external and internal disturbances identified.

#### Staff training

Investigate how staff education in sleep hygiene impacts residents’ experiences. As noted by Cap et al. (2024), trust in caregivers is pivotal for creating a restful environment.

#### Longitudinal designs

Track changes in sleep experiences over time, particularly in relation to life transitions (e.g., retirement and loss of loved ones) highlighted in the study.

### Future directions

To build on this study, future research should:

1. Incorporate mixed methods: Combining qualitative insights with objective sleep measures (e.g., actigraphy) could strengthen validity.
2. Address diversity: Expanding recruitment to include more men and cognitively impaired residents would improve representativeness.
3. Investigate interventions: Testing tailored strategies, such as noise-reduction protocols or staff education programs, could bridge the gap between understanding and action.

## Conclusion

While Telhede’s methodology effectively captures the complexity of insomnia in nursing homes, addressing the above limitations could enhance the study’s rigour and applicability. Future research should prioritize multidisciplinary collaboration, inclusive sampling, and integrated interventions to bridge the gap between understanding and improving sleep for older adults in institutional settings.

## Supplementary Material

R1letter.docx
