# Supplementary material for: Critical evaluation of methodology in “Experiences of insomnia among older people living in nursing homes a qualitative study”
Source: Int J Qual Stud Health Well-being. 2025 Jun 27;20(1):2522442. doi: 10.1080/17482631.2025.2522442 (PMC12207777; doi:10.1080/17482631.2025.2522442)
Supplement: R1letter.docx [file ZQHW_A_2522442_SM9338.docx]

Letter to the Editor

Critical Evaluation of Methodology in “Experiences of insomnia among older people living in nursing homes A qualitative study”

Milad Kazemi najm^1^, Nasrin Imanifar^2,3^

1. PhD student in nursing, Student Research Committee, Hamadan University of Medical Sciences, Hamadan, Iran. Email: [m.kazeminajm@edu.umsha.ac.ir](mailto:m.kazeminajm@edu.umsha.ac.ir) Orcid: <https://orcid.org/0000-0002-8159-2049>
2. MSc of Nursing, Instructor and Faculty Member of the Department of Nursing, Khor.C., Islamic Azad University, Khorramabad, Iran.
3. PhD student in nursing, Student Research Committee, Nursing and Midwifery Faculty, USERN Office, Lorestan University of Medical Sciences, Khorramabad, Iran. Email: [nasrinimanifar@bums.ac.ir](mailto:nasrinimanifar@bums.ac.ir) Orcid: <https://orcid.org/0000-0001-7176-0849>

Dear Editor in chief,

Professor Henrika Jormfeldt

We read with interest the recent article by Eva Hjort Telhede (2025) exploring the experiences of insomnia among older people in nursing homes. This qualitative study provides valuable insights into the multifaceted nature of sleep disturbances in this vulnerable population (Hjort Telhede, 2025). Below, I offer a critical evaluation of the research, highlighting its strengths, limitations, and implications for future work.

***Abstract:***

***Introduction:*** This letter critically evaluates the methodology of Eva Hjort Telhede’s qualitative study exploring insomnia experiences among nursing home. While the study contributes valuable insights into subjective sleep challenges, its methodological rigor warrants scrutiny to inform future research.

***Methods:*** The study employed a qualitative descriptive design with semi-structured interviews (n=19 participants) and inductive content analysis. Data collection occurred in nine Swedish nursing homes, with purposive sampling based on insomnia criteria (ICD-10) and cognitive competence (S-MMSE ≥ 20). Analysis followed Graneheim and Lundman’s qualitative content analysis framework.

***Results:*** Key methodological strengths included purposive sampling, data saturation, and reflexive practices. Limitations identified were single-researcher bias, lack of intercoder reliability checks, gender imbalance (4 men, 15 women), and exclusion of variables such as cognitive diagnoses and medication use. Environmental factors (e.g., noise, lighting) were self-reported without objective validation, and contextual transferability was constrained by limited demographic diversity.

***Discussion:*** The reliance on a single coder and absence of triangulation may compromise the depth of thematic analysis. Recommendations include: (1) multi-researcher collaboration to enhance credibility; (2) inclusive sampling of residents with dementia; (3) mixed-methods designs integrating objective sleep measures; and (4) staff training in sleep hygiene to address institutional barriers. Strengthening methodological transparency and addressing contextual factors could improve future interventions for insomnia in nursing homes.

***Keywords:*** Qualitative research; Methodology critique; Nursing homes; Insomnia; Content Analyzes, Older People

***Strengths***

The study’s qualitative design effectively captures the subjective lived experiences of insomnia, emphasizing the emotional, psychological, and environmental dimensions often overlooked in quantitative approaches. By employing semi-structured interviews and inductive content analysis, the author adeptly identifies two overarching categories—“Valuing good sleep” and “Disturbing influence on sleep”—with subcategories that align with existing literature on insomnia in older adults (e.g., anxiety, environmental noise, and social isolation) (Hjort Telhede, 2025). The purposive sampling strategy, grounded in ICD-10 criteria and cognitive competence (via S-MMSE), strengthens the validity of participant selection, ensuring relevance to the study’s aims.

The inclusion of direct participant quotes (e.g., “If you don’t do anything during the day, it’s boring...”) enriches the findings, offering authentic voices to contextualize the data. Furthermore, the author’s reflexive approach—acknowledging potential biases and refining interview questions through a pilot test—enhances methodological transparency.

***Limitations and Areas for Improvement***

Despite its merits, the study has notable limitations.

***1. Single Researcher Bias*:**

The sole involvement of the author in data collection, analysis, and interpretation introduces potential bias. Lack of triangulation (e.g., peer debriefing, multiple coders) limits the rigor of theme identification. As noted in the study’s limitations, this could compromise the depth of analysis (Malterud, 2001).

***2. Sample Demographics:***

The sample includes only four male participants (21%), which may limit generalizability given documented gender differences in sleep patterns and insomnia prevalence (Chu, Oh, & Lee, 2022). Future studies should aim for balanced representation.

***3. Exclusion of Key Variables:***

The exclusion of residents with formal dementia diagnoses or detailed medication histories weakens the analysis of how cognitive impairment or polypharmacy might mediate sleep disturbances. These factors are critical in nursing home populations (Laver et al., 2020).

***4. Environmental Factors:***

While residents highlighted environmental disturbances (e.g., noise, lighting), the study relied solely on self-reports. Objective measures (e.g., noise sensors, actigraphy) could validate these claims and strengthen findings (Ogilvy Dunstan et al., 2025).

***5. Lack of Intercoder Reliability:***

The absence of intercoder reliability checks undermines the trustworthiness of the analysis. Independent coding by multiple researchers would enhance confirmability (Elo & Kyngäs, 2008).

***6. Contextual Transferability:***

Findings are context-specific to Swedish nursing homes. The lack of data on staff-to-resident ratios, care routines, or facility size limits transferability to other cultural or institutional settings (Jung, Kim, & Choi, 2022).

***Discussion and Recommendations***

The discussion adeptly links findings to prior research, such as the role of environmental factors (e.g., noise, lighting) in sleep quality (Yang et al., 2022). However, the authors could have engaged more critically with conflicting evidence—for example, studies suggesting that structured daytime activities improve sleep in nursing home residents (Kim & Yoon, 2020), which contrasts with the current finding that reduced activity exacerbates insomnia.

The conclusion’s call for person-centered care and environmental modifications is well-founded. However, the recommendations remain broad. Future work could explore targeted interventions, such as staff training in sleep hygiene (Čáp et al., 2024) or personalized light therapy to regulate circadian rhythms (Kim & Yoon, 2020). Non-pharmacological strategies like cognitive behavioral therapy for insomnia (CBT-I) or weighted blankets—referenced in the author’s prior work (Telhede, Arvidsson, & Karlsson, 2024)—deserve further empirical attention.

***Recommendations for Future Research***

***1. Triangulation:***

Future studies should involve multiple researchers in coding and analysis to mitigate bias. Peer debriefing and audit trails could further enhance rigor (Polit & Beck, 2008).

***2. Inclusive Sampling:***

Expand recruitment to include residents with mild cognitive impairment or dementia, as these groups are often underrepresented yet highly vulnerable to insomnia.

***3. Mixed-Methods Approach:***

Combine qualitative insights with objective sleep measures (e.g., actigraphy) and environmental assessments (e.g., noise levels) to contextualize self-reports.

***4. Intervention Studies:***

Explore non-pharmacological interventions (e.g., weighted blankets, cognitive behavioral therapy for insomnia [CBT-I], or light therapy) to address the external and internal disturbances identified.

***5. Staff Training:***

Investigate how staff education in sleep hygiene impacts residents’ experiences. As noted by Cap et al. (2024), trust in caregivers is pivotal for creating a restful environment.

***6. Longitudinal Designs:***

Track changes in sleep experiences over time, particularly in relation to life transitions (e.g., retirement, loss of loved ones) highlighted in the study.

***Future Directions***

To build on this study, future research should:

1. Incorporate mixed methods: Combining qualitative insights with objective sleep measures (e.g., actigraphy) could strengthen validity.

2. Address diversity: Expanding recruitment to include more men and cognitively impaired residents would improve representativeness.

3. Investigate interventions: Testing tailored strategies, such as noise-reduction protocols or staff education programs, could bridge the gap between understanding and action (Dew et al., 2020).

**Conclusion:**

While Telhede’s methodology effectively captures the complexity of insomnia in nursing homes, addressing the above limitations could enhance the study’s rigor and applicability. Future research should prioritize multidisciplinary collaboration, inclusive sampling, and integrated interventions to bridge the gap between understanding and improving sleep for older adults in institutional settings.

**Conflict of interest:** The authors declare that there are no potential conflicts of interest regarding this article’s investigation, composition, or publication.

**Funding:** The authors did not receive any financial support for research, preparation, or publication of this article.

***Availability of data and materials:*** not applicable as no data was generated.

Sincerely,

Milad Kazemi najm

Nursing Ph.D student

Hamadan University of Medical Sciences

***References***

Čáp, J., Miertová, M., Bóriková, I., Žiaková, K., Tomagová, M., & Gurková, E. (2024). Trust in healthcare professionals of people with chronic cardiovascular disease. *Nursing ethics, 31*(6), 1092-1105.

Chu, H. S., Oh, J., & Lee, K. (2022). The relationship between living arrangements and sleep quality in older adults: gender differences. *International Journal of Environmental Research and Public Health, 19*(7), 3893.

Elo, S., & Kyngäs, H. (2008). The qualitative content analysis process. *Journal of advanced nursing, 62*(1), 107-115.

Hjort Telhede, E. (2025). Experiences of insomnia among older people living in nursing homes A qualitative study. *International Journal of Qualitative Studies on Health and Well-being, 20*(1), 2476788.

Jung, S. O., Kim, H., & Choi, E. (2022). The Effects of Non-pharmacological Interventions on Sleep among Older Adults in Korean Long-term Care Facilities: A Systematic Review and Meta-analysis. *Research in Community and Public Health Nursing, 33*(3), 340-355.

Kim, D. E., & Yoon, J. Y. (2020). Factors that influence sleep among residents in long-term care facilities. *International Journal of Environmental Research and Public Health, 17*(6), 1889.

Laver, K. E., Spargo, C., Saggese, A., Ong, V., Crotty, M., Lovato, N., . . . Vakulin, A. (2020). Sleep disturbance and disorders within adult inpatient rehabilitation settings: a systematic review to identify both the prevalence of disorders and the efficacy of existing interventions. *Journal of the American Medical Directors Association, 21*(12), 1824-1832. e1822.

Malterud, K. (2001). Qualitative research: standards, challenges, and guidelines. *The lancet, 358*(9280), 483-488.

Ogilvy Dunstan, O. B., Hanjani, L. S., Rodriguez, F., Garcia-Hansen, V., Hubbard, R. E., Young, A., & Ellender, C. M. (2025). Scoping review of the measurement of care environment factors that impact sleep in the rehabilitation, subacute, and aged care settings. *Sleep Advances, 6*(2), zpaf011.

Polit, D. F., & Beck, C. T. (2008). *Nursing research: Generating and assessing evidence for nursing practice*: Lippincott Williams & Wilkins.

Telhede, E. H., Arvidsson, S., & Karlsson, S. (2024). Frail older adults with insomnia experiences of using weighted blankets: A qualitative study. *Nordic Journal of Nursing Research, 44*, 20571585241279973.

Yang, E., Ismail, A., Kim, Y., Erdogmus, E., Boron, J., Goldstein, F., . . . Zimring, C. (2022). Multidimensional environmental factors and sleep health for aging adults: a focused narrative review. *International Journal of Environmental Research and Public Health, 19*(23), 15481.
